# A prospective study of stomach cancer death in relation to green tea consumption in Japan

**DOI:** 10.1038/sj.bjc.6600487

**Published:** 2002-08-01

**Authors:** Y Hoshiyama, T Kawaguchi, Y Miura, T Mizoue, N Tokui, H Yatsuya, K Sakata, T Kondo, S Kikuchi, H Toyoshima, N Hayakawa, A Tamakoshi, Y Ohno, T Yoshimura

**Affiliations:** Department of Public Health, Showa University School of Medicine, 1-5-8 Hatanodai, Shinagawa, Tokyo 142-8555, Japan; Department of Nursing, Saitama University Saitama, Japan; Department of Clinical Epidemiology, Institute of Industrial Ecological Sciences, University of Occupational and Environmental Health, Fukuoka, Japan; Department of Public Health/Health Information Dynamics, Field of Social Life Science, Program in Health and Community Medicine, Nagoya University Graduate School of Medicine, Nagoya, Japan; Department of Public Health, Wakayama Medical University, Wakayama, Japan; Department of Public Health, Aichi Medical University, Aichi, Japan; Department of Epidemiology, Research Institute for Radiation Biology and Medicine, Hiroshima University, Hiroshima, Japan; Department of Preventive Medicine/Biostatistics and Medical Decision Making, Field of Social Life Science, Program in Health and Community Medicine, Nagoya University Graduate School of Medicine, Nagoya, Japan

**Keywords:** green tea, stomach cancer, JACC study

## Abstract

To evaluate whether green tea consumption provides protection against stomach cancer death, relative risks were calculated using Cox proportional hazards regression analysis in the Japan Collaborative Study for Evaluation of Cancer Risk, sponsored by the Ministry of Health and Welfare (JACC Study). The study was based on 30 370 men and 42 481 women aged 40–79. After adjustment for age, smoking status, history of peptic ulcer, family history of stomach cancer along with certain dietary items, the risks associated with drinking one or two, three or four, five to nine, and 10 or more cups of green tea per day, relative to those of drinking less than one cup per day, were 1.6 (95% CI: 0.9–2.9), 1.1 (95% CI: 0.6–1.9), 1.0 (95% CI: 0.5–2.0), and 1.0 (95% CI: 0.5–2.0), respectively, in men (*P* for trend=0.669), and 1.1 (95% CI: 0.5–2.5), 1.0 (95% CI: 0.5–2.5), 0.8 (95% CI: 0.4–1.6), and 0.8 (95% CI: 0.3–2.1), respectively, in women (*P* for trend=0.488). We found no inverse association between green tea consumption and the risk of stomach cancer death.

*British Journal of Cancer* (2002) **87**, 309–313. doi:10.1038/sj.bjc.6600487 www.bjcancer.com

© 2002 Cancer Research UK

## 

Stomach cancer is still the leading cause of cancer death among women and the second among men in Japan (Ministry of Health and Welfare Japan, 2000). It has recently been reported (Setiawan *et al*, 2001; Inoue *et al*, 1998) that the consumption of green tea is inversely associated with the risk of stomach cancer, that is a protective effect. Green tea polyphenols have various anticarcinogenic effects, such as strong antioxidant activity, inhibition of nitrosation and cell proliferation.

Although case-control studies (Setiawan *et al*, 2001; Inoue *et al*, 1998; Ji *et al*, 1996; Yu *et al*, 1995; Kono *et al*, 1988) have found a reduced risk of stomach cancer in association with the consumption of green tea, prospective studies (Tsubono *et al*, 2001; Galanis *et al*, 1998) have not. In two Japanese studies, a decreased risk of stomach cancer was associated with the highest level of consumption of green tea (10 or more cups per day in one study (Kono *et al*, 1988) and seven or more cups per day in the other (Inoue *et al*, 1998)) but not with intermediate levels of consumption. The present study aimed to examine prospectively the association between the consumption of green tea and the risk of stomach cancer death, while controlling potential confounders, using data from the Japan Collaborative Cohort (JACC) Study, a Japan-wide population-based prospective study. This is the first study to analyse prospectively the effects of the consumption of 10 or more cups of green tea per day.

## MATERIALS AND METHODS

### JACC study

The JACC Study, the Japan Collaborative Cohort Study for Evaluation of Cancer Risk (sponsored by the Ministry of Education, Science, Sports and Culture of Japan), is a nation-wide multicentre prospective study to evaluate various risks on cancer incidence and mortality. Study methods and ethical issues have been described in detail elsewhere (Ohno *et al*, 2001; Aoki, 1996; Subcommittee of ethical issues, 1996). Briefly, our study was initiated in 1988 and enrollment continued until the end of 1990. Subjects were followed until the end of 1997 unless they had moved or developed one of the prospectively defined endpoints. Forty-five municipalities were involved in this prospective study. They included six cities, 34 towns and five villages, which covered most of Japan. Enrollment was drawn from participants in the general health checkups that are periodically provided by Japanese municipalities. Because we estimated 1 000 000 person-years of follow-up was necessary for the detection of an association between mortality for cancer of several sites common in Japan and various risk factors, we enrolled 127 477 (54 032 men and 73 445 women) apparently healthy inhabitants after completion of a questionnaire. Two strategies were applied to obtain informed consent for participation; in the majority of study areas, it was obtained with a signature on the cover page of the questionnaire. In some study areas, it was obtained at group level by explaining the aim of the study and confidentiality of the data to the leader of the community (Yatsuya *et al*, 2002). The questionnaire was filled in by the participants at home and checked by interviewers. Missing and unclear answers were confirmed or corrected by telephone.

The research protocol of the present study was approved by the Ethics Committee of Medical Care and Research, University of Occupational and Environmental Health, Kitakyushu, Japan.

### Follow-up and identification of stomach cancer cases

Our primary endpoints were death from any causes on December 31, 1997 (censored). Those who had moved address were also treated as censored. The mean follow-up period was 8.0 years for men and 8.2 years for women.

A follow-up survey was conducted annually to verify the vital status of participants. For deceased subjects, the cause of death was obtained from the death certificate at the regional health center, with the permission of the Ministry of Public Management, Home Affairs, Post and Telecommunications. These data were collected at the central office of the Research Committee. Underlying causes of death were determined by the Ministry of Health, Labour and Welfare and coded according to the International Classification of Disease (9th revision: ICD-9) by the end of 1994 and to ICD-10 from 1995. The stomach cancer codes were 151.0 to 151.9 of ICD-9 or C16.0 to c16.9 of ICD-10.

### Subjects

Since questions on the daily consumption of green tea were not included in the questionnaire in seven areas (four rural areas and three urban/rural areas), we excluded those data (15 609 men and 19 894 women). Of the 91 974 remaining, the 6469 who were under 40, and the 3115 who were 80 or over were excluded from the analysis. Of the 82 390 participants who were aged 40–79 years (34 173 men and 48 217 women) at the time of enrollment, we excluded those with a history of stomach cancer (*n*=216) and those who were followed-up for 12 months or less (*n*=800). Of the remaining 33 612 men and 47 762 women aged 40 to 79, 3242 men (9.6%) and 5281 women (11.1%) had green tea consumption data missing from the questionnaire so these too were excluded. The remaining 30 370 men and 42 481 women were included in the present analysis (see Appendix I).

### Questionnaire

The self-administered questionnaire on baseline characteristics covered medical history and included lifestyle-related questions such as diet, physical activity, drinking and smoking, occupation, level of education, reproductive history (women only) and the family history of several medical conditions including cancer. Mean time spent filling out the questionnaire was 26.0 and 27.7 min for men and women, respectively.

Questions on dietary habits included the consumption frequency of food items/groups. Five frequency categories were determined for consumption of most dietary products. In addition, the questionnaire asked for an individual's preference for salty foods, consumption of nonalcoholic beverages, smoking, and alcohol. Information on the intake of energy and other nutrients was not available in this study. The frequency of green tea consumption was initially assessed from five possible answers, i.e., every day (more than one cup per day), three to four cups per week, one to two cups per week, one to two cups per month and seldom, to the question: ‘Do you drink Japanese tea (green tea)?’ For those who consumed tea every day, the number of cups a day was further identified. We created five categories based on the answers to these questions: less than one cup per day, one or two cups per day, three or four cups per day, five to nine cups per day, and 10 or more cups per day according to a previous prospective study (Tsubono *et al*, 2001) for the purpose of compatibility. In Japan the volume of a typical cup of green tea is 100–120 ml.

The validity of this food frequency questionnaire was evaluated by comparing it with four 3-day dietary records (total weight) for eight men and 77 women selected from the study areas. Spearman's correlation coefficients for the frequencies were 0.41 for spinach and other coloured vegetables, 0.45 for tomatoes and white vegetables, 0.35 for fruits, and 0.29 for green tea, while other foods ranged between 0.1 and 0.8 (unpublished data).

### Data processing

Cox proportional hazard regression analysis was used. The relative risk (RR) and its 95% confidence interval (CI) were calculated based on the regression coefficient and its standard error (Cox, 1972) for an indicator term corresponding to a level of independent variable. For multivariate analysis, several factors were listed as potential confounders according to epidemiological studies (World Cancer Research Fund, 1997; Yatsuya *et al*, 2002; Hoshiyama and Sasaba, 1992). The model always included age (four categories: 40–49, 50–59, 60–69, 70–79). Trends of association were assessed by the regression model assigning scores (0–4) to the levels of the independent variable. Statistical significance (two sided) was based on the ratio of the regression coefficient and its standard error. Statistical analysis (PHREG procedure) was performed by the Statistical Analysis System (Sas Institute, 1983).

## RESULTS

Table 1

**Table 1:**
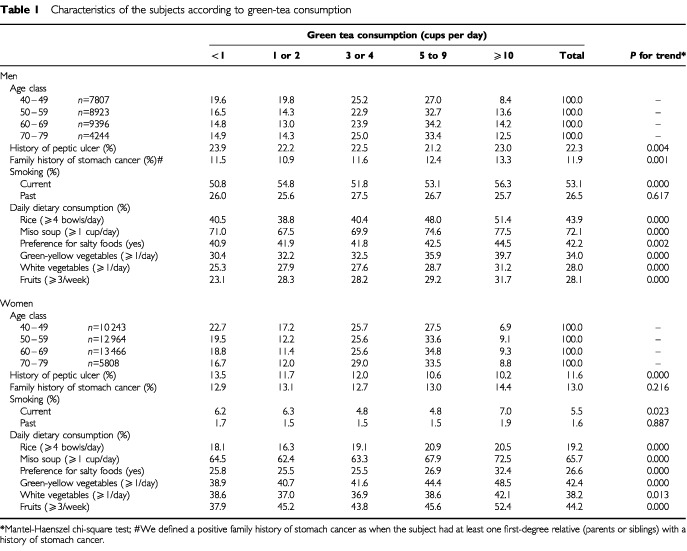
Characteristics of the subjects according to green-tea consumption

compares the characteristics of subjects according to green tea consumption, which showed a similar pattern in men and women. A history of peptic ulcer differed by green tea consumption in both men and women as examined by the Mantel-Haenszel chi-square test for trends (*P*<0.01), being higher among those who consumed less green tea. The proportions of smokers were higher among those who consumed more green tea for both men (*P*<0.01) and women (*P*<0.05). Men and women with a higher intake of green tea also tended to consume rice, miso soup, green-yellow vegetables, white vegetables and fruit more frequently.

Table 2

**Table 2:**
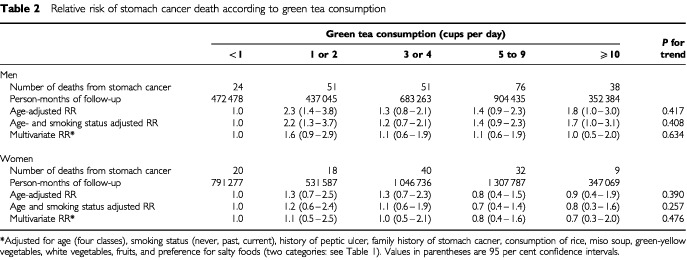
Relative risk of stomach cancer death according to green tea consumption

shows relative risk (RR) of stomach cancer and its 95% confidence interval (CI) according to green tea consumption, and *P*-value for trends. The age-adjusted relative risk of male subjects drinking one or two cups per day and 10 or more cups per day showed 2.3 (95% CI: 1.4–3.8) and 1.8 (95% CI: 1.0–3.0); however, after adjustment for certain dietary elements, history of peptic ulcer and family history of stomach cancer as potential confounders; this association was not statistically significant. No dose-response effect from heavier green tea consumption was observed (*P* for trend >0.05). Multivariate RRs were closer to age-adjusted and age- and smoking adjusted RRs in women. The exclusion of deaths from stomach cancer identified in the first 35 months of follow-up did not alter the findings (data not shown).

The association in the multivariate models between these potential confounders and stomach cancer was not statistically significant except for a family history of stomach cancer (RRs were 1.5 (95% CI: 1.0–2.3) for men and 2.6 (95% CI: 2.6–4.3) for women).

## DISCUSSION

This study is the largest prospective study to investigate green tea consumption and the risk of stomach cancer death. Among possible limitations of the present study was incomplete data. About 10% of subjects were excluded from analysis because they had not given information concerning their daily consumption of green tea and the effects of such exclusion are unknown. Nevertheless, there was no difference between the percentages of smokers in the excluded data (51.3% in men and 5.6% in women) and those in the included data (53.1% and 5.4% respectively) examined by Cochran-Mantel-Haenszel chi-square test (*P*=0.381 and *P*=0.068 respectively). The missing information seemed to occur randomly.

If drinking green tea, widely consumed in Japan and other Asian countries, protects against stomach cancer, it would be an inexpensive and convenient method of primary prevention. Another prospective study also found no association between green tea and stomach cancer (Tsubono *et al*, 2001). Little other evidence is available from prospective studies (Galanis *et al*, 1998). In the present study, we showed that high consumption of green tea (>=10 cups per day) was not associated with the risk of stomach cancer death based on multivariate analysis. We also showed that those with a higher intake of green tea also tended to consume rice, miso soup, green-yellow vegetables, white vegetables and fruits more frequently, and that a higher proportion of current smokers also consumed green tea more frequently. Another strong potentially limiting factor is the possibility of general over-reporting since subjects consuming 10 or more cups of green tea per day also reported consuming more of every item asked. If it were so, the effects of high consumption of green tea would be masked by misclassification. However, green tea consumption did not show a dose-response relationship and any preventive effects of green tea might not be substantial after adjustments for several potential confounders.

In general, findings from case-control studies often conflict with those from prospective studies (Bushman, 1998; World Cancer Research Fund, 1997; Blot *et al*, 1996). In these retrospective studies, some patients with stomach cancer might have decreased their consumption of green tea before the diagnosis because of their abdominal symptoms. This change in practice might have biased their recall of past intake in such a way that they underestimated their true consumption, resulting in spurious inverse associations (Tsubono *et al*, 2001). We agree that such bias could partly explain the inconsistent results seen between different study designs.

In Japan, tea is usually made in china pots with hot water (about 80°C) and not only are the first extracts consumed, but also the second and/or the third as well. The effective components of green tea such as polyphenols might be insufficient in the second and/or third extracts. If high consumption of green tea (>=10 cups per day) were protective against stomach cancer, the Japanese custom of drinking second/third extracts would be less effective in the prevention of stomach cancer.

Another possible limitation was that we did not obtain information on the presence or absence of a history of infection with *Helicobacter pylori,* a strong risk factor for stomach cancer (Asaka *et al*, 1997). The subjects with chronic gastritis caused by *H. pylori* infection might have limited their consumption of green tea. If so, the prevalence of infection would have been lower in the subjects with higher intakes of green tea. If not, it is unlikely that the prevalence of infection is higher among the subjects with a high consumption of green tea. Thus, we believe that not considering *H. pylori* infection would not have masked an inverse association between the risk of stomach cancer death and the consumption of green tea.

In summary, we found no inverse association between the consumption of green tea and the risk of stomach cancer death in Japan in a prospective cohort study.

## JAPAN COLLABORATIVE COHORT STUDY GROUP

The present investigators involved in the JACC study and their affiliations are as follows: Dr Yoshiyuki Ohno, (the present chairman of the Monbusho ECC), Dr Akiko Tamakoshi (Secretary General of the Monbusho ECC), and Dr Hideaki Toyoshima, Nagoya University Graduate School of Medicine; Dr Mitsuru Mori, Sapporo Medical University School of Medicine; Dr Yutaka Motohashi, Akita University School of Medicine; Dr Shigeru Hisamichi, Tohoku University Graduate School of Medicine; Dr Yosikazu Nakamura, Jichi Medical School; Dr Takashi Shimamoto, Institute of Community Medicine, University of Tsukuba; Dr Haruo Mikami, Chiba Cancer Center; Dr Shuji Hashimoto, School of Health Sciences and Nursing, University of Tokyo; Dr Yutaka Inaba, Juntendo University School of Medicine; Dr Heizo Tanaka, Medical Research Institute, Tokyo Medical and Dental University; Dr Yoshiharu Hoshiyama, Showa University School of Medicine; Dr Hiroshi Suzuki, Niigata University School of Medicine; Dr Hiroyuki Shimizu, Gifu University School of Medicine; Dr Shinkan Tokudome, Nagoya City University Medical School; Dr Yoshinori Ito, Fujita Health University School of Health Sciences; Dr Akio Koizumi, Graduate School of Medicine and Faculty of Medicine, Kyoto University; Dr Takashi Kawamura, Kyoto University Center for Student Health; Dr Yoshiyuki Watanabe, Kyoto Prefectural University of Medicine, Research Institute for Neurological Diseases & Geriatrics; Dr Masahiro Nakao, Kyoto Prefectural University of Medicine; Dr Takaichiro Suzuki, Research Institute, Osaka Medical Center for Cancer and Cardiovascular Diseases; Dr Tsutomu Hashimoto, Wakayama Medical University; Dr Takayuki Nose, Tottori University Faculty of Medicine; Dr Norihiko Hayakawa, Research Institute for Radiation Biology and Medicine, Hiroshima University; Dr Takesumi Yoshimura, Institute of Industrial Ecological Sciences, University of Occupational and Environmental Health, Japan; Dr Katsuhiro Fukuda, Kurume University School of Medicine; Dr Tomoyuki Kitagawa, Cancer Institute of Japanese Foundation for Cancer Research; Dr Toshio Kuroki, Institute of Molecular Oncology, Showa University; Dr Naoyuki Okamoto, Kanagawa Cancer Center; Dr Teruo Ishibashi, Asama General Hospital; Dr Hideo Shio, Shiga Medical Center and Dr Kazuo Tajima, Aichi Cancer Center Research Institute.

 The former investigators involved in the JACC study and their affiliations are as follows: Dr Kunio Aoki, Aichi Cancer Center; Dr Suketami Tominaga, Aichi Cancer Center Research Institute; Dr Sadamu Anzai, Dr Takeshi Kawaguchi, Dr Kenichi Nakamura, Dr Motofumi Masaki, Showa University School of Medicine; Dr Shuugo Kanamori, Dr Masachika Morimoto, Dr Seishi Yoshimura, Shiga Medical Center for Adults; Dr Sigetosi Kamiyama, Dr Yukio Takizawa, Dr Noriyuki Hachiya, Akita University School of Medicine; Dr Keiichi Kawai, Dr Shuichi Nakagawa, Dr Hiroki Watanabe, Kyoto Prefectural University of Medicine; Dr Minoru Kurihara, Research Institute for Radiation Biology and Medicine, Hiroshima University; Dr Yoshio Komachi, Institute of Community Medicine, University of Tsukuba; Dr Ruichiro Sasaki, Aichi Medical University; Dr Minoru Sugita, Toho University School of Medicine; Dr Iwao Sugimura, Asahikawa Kosei Hospital; Dr Toshihiko Tanaka, Chigasaki Public Health Center; Dr Tomio Hirohata, Kyushu University School of Medicine; Dr Isaburo Fujimoto, Center for Adult Diseases, Osaka; Dr Minoru Matsuzaki, Chigasaki Public Health and Welfare Center; Dr Hirotsugu Miyake, Sapporo Medical University School of Medicine; Dr Motoi Murata, Chiba Cancer Center; Dr Shinsuke Morio, Kanagawa Cancer Center; Dr Hiroshi Yanagawa, Jichi Medical School and, Dr Shaw Watanabe, Tokyo University of Agriculture

## Figures and Tables

**Table 3:**
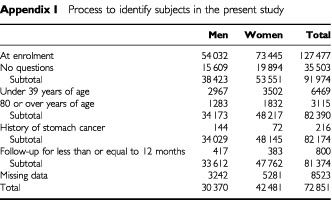
Process to identify subjects in the present study

## Appendix 1

Table 3
